# Supplementary material for: An integrative approach to prioritize candidate causal genes for complex traits in cattle
Source: PLoS Genet. 2025 May 30;21(5):e1011492. doi: 10.1371/journal.pgen.1011492 (PMC12158001; doi:10.1371/journal.pgen.1011492)
Supplement: Attachment — Submitted filename: PLOS review.pdf [file pgen.1011492.s007.pdf]

Writing needs to be improved. Additional details on the animals and conditions associated with the different data sets are needed. Additional details on models used for the different analyses are needed, specifically, information on fixed effects. See detailed comments.

Linkage disequilibrium is referred to as an issue in the abstract but is not referred to at all in the discussion. If it's an issue, should it not be discussed? Also, how does LD relate to the choice of window size when identifying GWAS-Marked windows?

L17 "identify the gene through which QTL act". Poor wording, as it could imply to refer to identifying genes from which the QTL is an eQTL, rather than the gene that harbors the causative variant.

L41 "The overlap between GWAS, GSOR, and GO analyses suggests that together these methods can identify genes mediating QTL, though their power remains limited, as reflected by modest odds ratios." I think this statement is misleading because none of these methods, either individually or combined can identify the causative gene for a QTL with certainty. I.e. there will be many false positives. The odds ratio points to the probability that the causative gene is among those that are identified, but it may not be and, if multiple genes are identified, there's no information to identify the correct gene.

L44 'issues of linkage disequilibrium' are not referred to at all in the discussion. This should be discussed.

L52 "To identify the gene through which QTL work" Poor wording – see above.

L85 Another way to identify genes whose expression is (genetically) related to a phenotype is to estimate genetic correlations between gene expression and the trait (see e.g. Lim et al. 2023. GSE *55*(1), p.90)

L120 Methods do not describe details on significance testing in individual-trait GWAS analyses.

L158 What are GWAS-Marked windows?

L202 "Several factors contribute to TWAS cause false positives" Poor wording.

L283 Additional details are needed on the choice of cows. Were they all from the same herd? The same parity? Were they genetically related?

L298 Effects included in the model need to be specified. Also assumptions about variance-covariance structures.

L300 How many were excluded for this reason?

L301 How were the cows genotyped with different platforms chosen?

L308 How were these chosen and what were their features (herd, parity, DIM, etc). Context is important, especially for gene expression data.

L311 Imputation based on genotypes derived from the RNAseq data?

L313 Same questions as for the Australia gene expression data

L323 There were no systematic factors that needed to be included as fixed effects?

L333 What's the hypothesis that this statistic is designed to test?

L353 So here you are including batch by year as a fixed effect? A description of batch and year needs to be given in the section that describes the phenotype recording

L370 You need to describe the fixed effects included.

L376 Need details on 'experiment' and 'DIM'.

L377 "Fixed effects were not available for the mammary RNA-seq dataset." So there were fixed effects that should have been fitted but you did not have the necessary information? That's a bit troubling.

L380 Using all p-values across all genes and traits as the reference set?

L389 You used either 100 or 500 kb?
