# Supplementary material for: An integrative approach to prioritize candidate causal genes for complex traits in cattle
Source: PLoS Genet. 2025 May 30;21(5):e1011492. doi: 10.1371/journal.pgen.1011492 (PMC12158001; doi:10.1371/journal.pgen.1011492)
Supplement: Attachment — Submitted filename: Response to Reviewers.docx [file pgen.1011492.s009.docx]

PGENETICS-D-24-01281
An integrative approach to prioritize candidate causal genes for complex traits in cattle
PLOS Genetics

**Reviewers' comments:**

Reviewer #1:

Writing needs to be improved. Additional details on the animals and conditions associated with the different data sets are needed. Additional details on models used for the different analyses are needed, specifically, information on fixed effects. See detailed comments.

R: Thank you for your comments. We improved writing (Please note that all RED highlights in the manuscript are modifications made to improve written language, and the BLUE ones are modifications made to address the reviewers’ comments)

Linkage disequilibrium is referred to as an issue in the abstract but is not referred to at all in the discussion. If it’s an issue, should it not be discussed? Also, how does LD relate to the choice of window size when identifying GWAS-Marked windows?

R: We already discussed LD in lines 208 and 222.

R: We tested two window sizes to account for LD, and we got better results for the shorter windows. We don’t see any value in going over 500 Kb (please see Tables 3 and 4).

L17 “identify the gene through which QTL act”. Poor wording, as it could imply to refer to identifying genes from which the QTL is an eQTL, rather than the gene that harbors the causative variant.

R: In the discussion (line 204-205) we already mentioned that “The causal variant may directly point to the associated gene if it is located in a coding region,” but in this sentence, we intend to refer to identifying genes from which the QTL is an eQTL.

L41 “The overlap between GWAS, GSOR, and GO analyses suggests that together these methods can identify genes mediating QTL, though their power remains limited, as reflected by modest odds ratios.” I think this statement is misleading because none of these methods, either individually or combined can identify the causative gene for a QTL with certainty. I.e. there will be many false positives. The odds ratio points to the probability that the causative gene is among those that are identified, but it may not be and, if multiple genes are identified, there’s no information to identify the correct gene.

R: Revised (Line 42).

R: We already addressed the issue of the identification of multiple genes in the discussion (lines 222-235)

L44 ‘issues of linkage disequilibrium’ are not referred to at all in the discussion. This should be discussed.

R: R: We already discussed LD (please see lines 208 and 222 in the revised version).

L52 “To identify the gene through which QTL work” Poor wording – see above.

R: In this specific sentence, we intend to refer to identifying genes from which the QTL is an eQTL, but also, in the discussion (lines 204-205), we mentioned: “The causal variant may directly point to the associated gene if it is located in a coding region.”

L85 Another way to identify genes whose expression is (genetically) related to a phenotype is to estimate genetic correlations between gene expression and the trait (see e.g. Lim et al. 2023. GSE 55(1), p.90)

R: Revised accordingly, and the reference included (Line 92-93). GSOR uses a form of genetic correlation where it uses separate animals to estimate trait EBVs and gene expression. It has the advantage over a conventional genetic correlation because it uses only the SNPs in the vicinity of the gene to estimate the trait EBV and thus gains in power.

L120 Methods do not describe details on significance testing in individual-trait GWAS analyses.

R: Done! Lines 381-384

L158 What are GWAS-Marked windows?

R: We addressed this issue by adding an explanation immediately after the term, referring readers to the Methods section (line 178).

L202 “Several factors contribute to TWAS cause false positives” Poor wording.

R: Revised (line 222).

L283 Additional details are needed on the choice of cows. Were they all from the same herd? The same parity? Were they genetically related?

R: We added some details regarding the sampling process (lines 307-320). We checked the pedigree file, based on which these cows represented 78 sires and 265 dams. However, we fitted a GRM in the GCTA analyses so correcting for relatedness.

L298 Effects included in the model need to be specified. Also assumptions about variance-covariance structures.

R: Done! (lines 334-341)

L300 How many were excluded for this reason?

R: Done! Please see lines 343-345.

L301 How were the cows genotyped with different platforms chosen?

R: The cows had already been genotyped for other project work (Ellinbank experimental herd). Please see lines 346-347.

L308 How were these chosen and what were their features (herd, parity, DIM, etc). Context is important, especially for gene expression data.

R: We added a sentence to the text to address this question (lines 337-339). Information regarding DIM and sampling time was also added to the text. Pleas e see Methods section, the GSOR subsection (lines 417-420).

L311 Imputation based on genotypes derived from the RNAseq data?

R: Revised. Line 361 >>> (Imputation to WGS genotypes for the WBC RNA-seq animals was conducted using Run7 of the 1000 Bull Genomes project …)

L313 Same questions as for the Australia gene expression data

R: The New Zealand (NZ) gene expression data was obtained from Livestock Improvement Corporation (LIC) NZ, and it was already imputed to the WGS level using their own reference population, as illustrated in the text (lines 365).

L323 There were no systematic factors that needed to be included as fixed effects?

R: We revised (see lines 372-375) the sentence describing the model: which now reads: “GCTA initially adjusted the phenotypes for fixed effects (the combined effects of batch and year with nine levels), and then uses the following model: $\mathbf{y} = \mathbf{1}\mu+\mathbf{bx}+\boldsymbol{g}^{\boldsymbol{-}}+\mathbf{e}$(1), where $\mathbf{y}$ is a vector of records for adjusted phenotypic values …”

L333 What’s the hypothesis that this statistic is designed to test?
R: We already discussed this. Please see line 215 in the discussion section.

L353 So here you are including batch by year as a fixed effect? A description of batch and year needs to be given in the section that describes the phenotype recording

Revised: Please see lines 297-301.

L370 You need to describe the fixed effects included.
R: In lines 427-443, we first described the model, and then we explained the fixed effects included regarding the two gene expression dataset.

L376 Need details on ‘experiment’ and ‘DIM’.

R: Revised! Please see lines 436-439.

L377 “Fixed effects were not available for the mammary RNA-seq dataset.” So, there were fixed effects that should have been fitted but you did not have the necessary information? That’s a bit troubling.

R: Revised! Lines 438-439.

L380 Using all p-values across all genes and traits as the reference set?
R: Revised. We added: “…, conducted separately for each trait.” (line 442)

L389 You used either 100 or 500 kb?

R: Yes, we used both.

Reviewer #2: This is a very interesting, clear and well written article. The main originality is the use of the GSOR method. However, this method has already been described in a paper (by the same team) deposited on BioRxiv, and therefore presumably submitted for publication.

Note: The BLUE highlights in the revised manuscript are modifications made to address the reviewers’ comments (and the red highlights are made to improve the written language as one of the reviewers requested this)

R: Thank you for your comments. The GSOR method paper is currently under review in another scientific journal, and due to extensively prolonged peer-review process, it has not yet been published.

If I understand correctly, the present article is therefore an application of this method. For a good demonstration, the GWAS dataset does not seem to me to be the most appropriate. Of course, the traits are selected for their assumed simple determinism, which limits the number of expected QTL. But the limited size of the dataset does not prove the universal effectiveness of the method: GWAS have limited power and the partial genomic values are likely to have very low accuracy. For a more convincing demonstration, I would have preferred to see a larger application.

R: Thank you for your feedback. We already addressed this issue as a limitation of our study in the discussion section (lines starting from 289)

R: We do not claim that our findings are universally true. Instead, we have presented results based on the current dataset, which are statistically significant. Unfortunately, it is not feasible to increase the sample size for these traits.

Note: An important determinant of polar lipids in milk is the size of the fat globules. The smaller they are, the more membranes there are. I think this point could be discussed. And fat globule size is much easier to measure than polar lipids. This could also explain the influence of genes that affect fatty acids, such as DGAT1.

R: We added this in the discussion section (lines 261-263).

Minor remarks
23: Do you mean : A multi-trait meta-analysis of GWAS… ?

R: Revised! Line 23

117: standard errors of h2 estimates seem quite optimistic with regards to the number of records

R: We believe the standard errors are reasonable because they are based on genotypes not pedigrees, and cattle have small effective population size and PL traits have simpler genetic architecture.

123: because Methods are presented after Results, it would be useful to define GWASMeta

R: We addressed this issue by adding an explanation immediately after the term, referring the reader to the Methods section (line 178-179).

367: I do not understand g in equation (4). Why explaining partial genomic values by whole genomic values?

R: In the equation (4), *b1* is the regression coefficient of local GEBV on gene expression. We used *g* in this equation to account for and remove the potential correlation (if it exists) between local GEBV and genome-wide GEBV.

Reviewer #3: Ghoreishifar et al. conducted a GWAS for 56 traits related to polar lipids in milk, utilizing three main sources of information: GWAS (including meta-GWAS hits), genetic score omics regression (GSOR), and GO analysis. Although the sample size for the GWAS is relatively small, some of the studied phenotypes are expected to exhibit simple genetic architectures, making it possible for the study to identify meaningful associations.

Note: The BLUE highlights in the revised manuscript are modifications made to address the reviewers’ comments (and the red highlights are made to improve the written language as one of the reviewers requested this)

R: Thanks for your comments.

A major concern, however, is that the GWAS, meta-GWAS, and GSOR analyses are all derived from the same dataset, which means these sources are not independent, potentially limiting the robustness of the findings.
R: We used independent data for GWAS and GSOR. Indeed, the mammary gene expression data was from NZ cows; we predicted GEBVs of PL using a BayesR model and an independent reference population (i.e., Australian GWAS data) for those NZ cows.

Regarding the GSOR methodology, the manuscript (overlapping authors with the current manuscript) has been available on bioRxiv since July 15, 2022. Wondering why it has not yet been published in a peer-reviewed journal.

R: Thank you for your comments. The GSOR method paper is currently under review in another scientific journal, and due to extensively prolonged peer-review process, it has not yet been published.

Line 62: It is worth noting that the association is not only on the same chromosome but also that the variant and the gene are closely located.

R: Revised! Lines 77-79

Line 93. “these traits are simpler” -> these traits are expected to have simpler genetic architecture

R: Revised! Lines 111-112

Line 121: How was this P-value threshold reached?

R: This p-value threshold is based on FDR 10%

Line 123-123. Did you examine how many independent QTL are segregating on these 2 chromosomes?

R: We did COJO analyses using GCTA and found five SNPs (P_Joint_ ≤ 1E-8) across 20 individual PL phenotypes, of which two were on BTA24, and three on BTA14 (Below is the results attached in a Table!). However, these significant SNPs were obtained across multiple PL phenotypes (GWAS). Thus, two possibilities include: (1) there is only one SNP in each region affecting multiple PLs, and (2) there are multiple SNPs, each affecting a specific type of PLs.

Line 129-130: did you run multi-trait meta-analysis for 21 PLs having ‘significant association’ in single-trait analysis?

R: As mentioned in the paper (lines 143 and 146-149), we run multi-trait meta-analyses using all the 56 PL phenotypes.

Line 232-233: “Seven of the 70 mammary GSOR hits located within GWAS-Marked windows contained the lipid metabolism GO term” – could you comment what are the other possible physiological mechanisms linking to PLs for the rest of GSOR hits (90%).

R: We found several GO terms significantly enriched by GSOR gene lists (see Table 2). However, they were not directly related to PL, and the overlapping analyses between GWAS and GSOR could not find any significant term other than polar lipids. They could potentially be false positives. In the discussion, we already addressed this point, which reads “not all gene-trait associations are causal. TWAS methods (including GSOR) often detect multiple significant genes per locus …”

Line 239-241. “The DGAT1 gene has been reported to account for 30-40% of the phenotypic variance of milk yield and composition in cattle [23, 24]”. Please check it carefully; r^2 (QTL) is 0.18 for milk yield (Grisart et al. 2002).

R: Thank you for your comment. We revised the sentence (line 259-261).

L317-330: The genomic inflation factors (lambda values) for GWAS should be presented (maybe as supplemental table), as LOCO approach can increase genomic inflation (<https://doi/10.1111/jbg.12419>).

R: Lambda were calculated for GWAS and presented in Additional file 1. It ranged from 0.94 to 1.07, with an average of 1.01. We added a description in the Methods (lines 385-389), and a sentence in the Results (lines 141-142).

L331-342: Multi-trait meta-analysis: There are two issues. The LD score regression approach (<https://doi.org/10.1038/ng.3406>) should be used for estimation of genetic correlation to avoid bias due to sample overlap and spread of LD. For similar reasons, i.e. the GWAS was done in one population for all the traits, approach like MTAG (<https://doi.org/10.1038/s41588-019-0469-9>) . should be employed.

R: The LD score regression doesn’t work in cattle because of the extensive LD. We tried them and it was useless.

R: The multi-trait meta-analyses method we used has been published and been used many times and we don’t see that there is a problem with it.
