# Supplementary material for: An integrative approach to prioritize candidate causal genes for complex traits in cattle
Source: PLoS Genet. 2025 May 30;21(5):e1011492. doi: 10.1371/journal.pgen.1011492 (PMC12158001; doi:10.1371/journal.pgen.1011492)
Supplement: Attachment — Submitted filename: Response to reviewers R2.docx [file pgen.1011492.s010.docx]

The major shortcomings are that the GWAS population is small and large number of traits (56) were analyzed. The manuscript describing the GSOR method is yet to be published as a peer-reviewed article. The results observed cannot be generalized as “comparatively simple series of traits” were selected.

R: We already acknowledge the small size of GWAS data as a limitation in our study (please see lines starting from 289) but despite this we report statistically significant and useful results. We do not have an option to increase sample size.

The GSOR manuscript is close to publication and is available on bioRxiv.

We do not argue that the results would necessarily apply to all traits. Any paper would be limited to the traits studied. We chose 56 PL traits because we argued that that represent a favourable case in which the physiology is well enough known to judge whether or not the analysis makes sense. And we show that genes found by GSOR and GWAS are enriched for lipid pathways. However, even in this case we do not find convincing pathways to explain all of the significant genes for PL traits which should act as a warning for the study of less well understood traits.

“As mentioned in the paper (lines 143 and 146-149), we run multi-trait meta-analyses using all the 56 PL phenotypes.”
Is it reasonable to assume the same genetic variant is affecting all 56 traits?

R: We discussed that multi-trait meta-analysis approach was used to analyse 56 milk PL traits because meta-analyses can enhance the power to detect genetic variants by leveraging the shared genetic architecture across correlated traits. The method does not assume that the same variant affects all traits: it tests the null hypothesis that the variant does not affect any of the traits and gains power by combining traits. However, it is true that variants tend to affect multiple traits or none.

“Line 232-233: “Seven of the 70 mammary GSOR hits located within GWAS-Marked windows contained the lipid metabolism GO term” – could you comment what are the other possible physiological mechanisms linking to PLs for the rest of GSOR hits (90%).
R: We found several GO terms significantly enriched by GSOR gene lists (see Table 2). However, they were not directly related to PL, and the overlapping analyses between GWAS and GSOR could not find any significant term other than polar lipids. They could potentially be false positives.”
If that is the case it seems too many false positives!

R: All three types of analysis generate false positives. In GWAS it is due to LD between the causal variant and nearby variants; in GSOR it is because of LD between a QTL and an eQTL for the wrong genes; in analysis of annotations such as GO terms it is due to imperfect knowledge of the multiple roles a gene plays especially in species other than humans. We show that by combining all three analyses we do identify good candidate genes. However, a conclusion of the paper is that we also identify genes with no **known** role in the trait.

“R: The LD score regression doesn’t work in cattle because of the extensive LD. We tried them and it was useless.
R: The multi-trait meta-analyses method we used has been published and been used many times and we don’t see that there is a problem with it.”
It is a very strange response. The second statement implies that a published article cannot be questioned. Contrary “We tried them, and it was useless” is very vague without substantiating.

We apologise for the brevity of our response. The paper does not use LD score regression and so it would be a diversion to discuss it at length. The reason LD score regression does not work in cattle is the widespread, long distance LD. This means the almost all variants have high LD scores and the differences between them do not reflect the probability that a variant is in LD with a causal variant. We have tried it and concluded that LD score regression is inappropriate for cattle but that is not the burden of this paper.

The reviewer is correct we could have used a multi-trait GWAS on the raw data, but the meta-analysis method gives good results and is easier to implement.
